# Socio-demographic and food insecurity associated with adherence to antiretroviral therapy among HIV adults in Ahmadu Bello University teaching hospital Zaria, Kaduna State Nigeria

**DOI:** 10.4314/ahs.v23i4.26

**Published:** 2023-12

**Authors:** Rosemary Ada Eze, Norhasmah Sulaiman, Zulfitri 'Azuan Mat Daud, Aliyu Babadoko

**Affiliations:** 1 Department of Nutrition, Faculty of Medicine and Health Sciences, Universiti Putra Malaysia, 43400 UPM, Serdang Selangor, Malaysia; 2 Department of Dietetics, Faculty of Medicine and Health Sciences, Universiti Putra Malaysia, 43400 UPM, Serdang Selangor, Malaysia; 3 Department of Haematology, Ahmadu Bello University Teaching Hospital, PMB 06, Zaria, Kaduna State, Nigeria

**Keywords:** Food insecurity, Socio-demographic, Antiretroviral therapy, HIV, Nigeria

## Abstract

**Background:**

Socio-demographic characteristics may have an impact on HIV-positive people's prognosis and survival. In addition, food insecurity could make it harder for HIV patients to stick to their treatment plans as effectively as possible.

**Objective:**

This research aimed to determine the association between socio-demographic and food insecurity with adherence to antiretroviral therapy among people living with HIV on ART in Ahmadu Bello University Teaching Hospital Zaria, Kaduna State, Nigeria.

**Method:**

Using a cross-sectional design, a systematic random sampling method was used to select respondents aged 18–64 years on antiretroviral therapy for at least six months at Ahmadu Bello University Teaching Hospital Zaria, Kaduna State, Nigeria, outpatients of the President's Emergency Plan for AIDS Relief clinic. Socio-demographic, food insecurity status and adherence to ART was obtained using self-administered questionnaire. Descriptive statistics, chi-square test, and multiple logistic regression were used for data analysis.

**Results:**

Among the 385 people who took part in the study, about 67.5% of females and 32.5% of males took part in the survey, respectively. About 54% of adults adhered to ART. The majority of the respondents (87.0%) had a low level of food security. Food insecurity (OR = 1.2, *p* = <0.05), government employment (OR = 2.842, *p* = <0.01), self-employment (OR = 2.6, *p* = <0.001), and being divorced or widowed (OR = 2.0, *p* = <0.01) were all significantly associated with ART adherence.

**Conclusion:**

Food insecurity, employment, and marital status influenced adherence to ART. As a result, health care providers and HIV control programme implementers must continually emphasis and encourage strict adherence.

## Introduction

According to the United Nations, in terms of disease burden, Nigeria is placed second in sub-Saharan Africa, behind South Africa, and third internationally, behind India^1^. The regions most affected by human immunodeficiency virus (HIV) are East and South Africa^2^. In low and middle-income countries, poor antiretroviral therapy (ART) adherence leads to higher hospitalisation rates, higher health-care expenses, decreased performance, family and community disruption, and increased morbidity and mortality^3^. Patients' socioeconomic position, education, literacy, and other factors, as well as the treatment regimen, illness characteristics, patient-provider relationships, and clinical surroundings, all influence adherence, according to previous studies in Sub-Saharan Africa^4^,^5^,^6^.

Socio-demographic factors have been demonstrated to influence adherence to antiretroviral therapy (ART) in HIV-positive people. According to the findings of an Indian study, females were more committed to ART than males^7^. According to a study conducted in Nigeria, gender has a crucial role in medication adherence among HIV and Acquired Immunodeficiency Syndrome (AIDS) patients^8^. This could be due to the economic disadvantage of the feminine gender, as most women are housewives who are not permitted to work for a living. Furthermore, Mitiku et al. found that the age group of 20–30 years had a greater adherence rate 92.8% 9. Calder et al. claim that research in north-central Nigeria indicated that women of Gwari and Nupe ethnicity had a lower probability of medication adherence than women of “other” ethnic groups, primarily Igbo and Yoruba^10^.

Differences in cultural beliefs among ethnic groups might explain the link between ethnicity and adherence. According to Nachega et al., HIV-positive people who work are more likely to stick to their ART regimen than those who are unemployed^11^. Compliance with ART may be influenced by social and economic factors based on job or occupational position. Marital status was one of the variables connected to antiretroviral medication adherence. According to research by Agu et al. and Bello, single patients appeared to be more non-adherent than married individuals and this was ascribed to a breakdown in communication among healthcare workers^12^,^13^. Anyaike et al. also discovered that a patient's educational level had a significant impact on treatment adherence^14^. When compared to individuals with a college education, those with no formal education were the most prone to forget about their drugs. Treatment duration has been linked to both adherence and non-adherence in studies^15^,^16^,^17^. This demonstrates that significant exposure to ART leads to improved knowledge and understanding for applying ART into everyday lives.

Food insecurity and the HIV/AIDS have a direct association. Food insecurity aggravates malnutrition, increasing the risk of HIV infection. HIV infection leads to food instability since it lowers farm productivity, income, and health bills, leading to a loss of capabilities^18^. In the overall adult population of people living with HIV, food insecurity impedes adherence to ART in four ways, according to qualitative assessments. The first is people's fears of, or real experiences with, greater appetite while taking ART on an empty stomach. Second, it's the dread of, or real encounters with, ART side effects that are amplified in the absence of appropriate nourishment. Third, people must choose between receiving food and getting medications, forcing them to exchange pharmaceuticals for meals. Finally, people must choose between food and medicines, forcing them to exchange or barter medicines in order to get food or other supplies. Insolvable hunger with ART has been observed in qualitative research from a number of African countries, where it is thought to be a main cause in non-adherence to treatment^19^,^20^,^21^.

This explains that although food-insecure HIV patients have accessibility to life-saving antiviral drugs, they are unable to meet the ART's dietary and nutritional requirements at all times due to their restricted or non-existent access to sufficient and nutritious food. Food insecurity among HIV-positive individuals can lead therapy to be delayed or even stopped altogether. Food hardship may make it increasingly challenging for HIV patients to stick to their treatment regimens as well as they should.

Non-compliance is linked to a number of factors that vary depending on the context. Knowledge was linked to non-adherence in East Jerusalem^22^. Younger age, rural residency, and substance use were all important factors in a study conducted in Kenya^9^. Internal migration, limited counselling, and the incapacity of the spouse to provide guidance and assistance were all cited as reasons for non-adherence to ART in Malawi^23^. According to a literature review, various researches have been conducted in other regions of Africa, but just a handful in Nigeria, such as Bello et al. in Ilorin, Afe et al. in Southwest Nigeria, and Okunola et al. in Ile-Ife on socio-cultural factors influencing antiretroviral therapy adherence^13^,^24^,^25^.

However, no study has been done in Nigeria on socio-demographic and food insecurity associated with adherence to ART among HIV adults. ABUTH is one of the government hospitals with a large provision of ART treatment for HIV patients in Nigeria. Therefore, this study aimed to determine between socio-demographic and food insecurity with adherence to ART among adults at ABUTH.

## Materials and methods

### Study design and setting

The study was conducted at ABUTH, Zaria, Kaduna State, Nigeria. Kaduna state is one of the 36 states in Nigeria, with the capital being Kaduna. In Nigeria, the top five states with highest HIV prevalence ae Akwa lbom (5.5%), Benue (5.3%), Rivers (3.8%), Taraba (2.9%), Anambra (2.4%) and Kaduna (1.1)^26^. An 18-month programme to increase the number of people receiving antiretroviral therapy (ART) in nine Nigerian states was launched in April 2019 by the U.S. President's Emergency Plan for AIDS Relief (PEPFAR). Despite the COVID-19 pandemic, the programme resulted in 208,202 additional HIV-infected people receiving ART (including 97,387 additional people from March 31, 2019 to March 31, 2020 and an additional 110,815 additional people from April 2020 to September (2020)^27^. However, the present study location Kaduna state was not one of the nine states that was selected for the study. Using purposive sampling ABUTH was selected in order to determinants and prevalence of adherence to ART. The study was done at the HIV/PEPFAR clinic at ABUTH, Zaria, one of the 11 hospitals in Kaduna State, Nigeria, that give antiretroviral therapy to HIV patients. The study was conducted from June until September 2019.

A cross-sectional study with a quantitative approach was conducted at the PEPFAR clinic of ABUTH, Zaria, Kaduna State, Nigeria. All adults aged 18 to 64 years living with HIV on ART at ABUTH, Zaria, Kaduna State were invited to participate in the study; adults who were too sick due to an illness or mental issue throughout the research period, such as infectious disorders or Alzheimer's, were not considered eligible. The sample size was calculated to be 385 and a systematic random sampling technique was used. Ethical approval was obtained from ethical committees of Universiti Putra Malaysia (JKEUPM-2019-036) and ABUTH, Zaria, Kaduna State, Nigeria (ABUTHZ/G30/2019). A questionnaire in English was used to collect the information. A variety of demographic, food security, and ART compliance questionnaires were filled out by those who took part.

The study adapted a 6-item scale from the US Household Food Security Survey Module^28^ to measure food insecurity. The adapted scale asked how often in the preceding 30 days the individual had to cut meal size, eat less, feel hungry, skip a meal, or go without food for the entire day because the household did not have enough money for food. “A lot,” “occasionally,” “never,” and “don't know” were among the options. Raw food insecurity scores [0–5] were created by adding positive responses (“a lot” or “sometimes”), which were then divided into three levels based on USDA classifications: “marginal food security or better” (0–1), “poor food security” (2–3), and “extremely low food security” (4–5) 29. However, in this study food security was categories into two level: food security (0-1) and food insecurity (2-6) see (Table 2).

The Centre for Adherence Support Evaluation (CASE) of the New York Academy of Medicine (NYAM) developed the CASE Adherence tool (CASE). CASE Adherence tool are three adherence questions in all. Better adherence is indicated by a higher composite score. The results of the CASE adherence instrument were combined to produce a combined measure that varied from 3 to 16 points. Non-adherent patients had an index score of less than ten, while those with a score of ≥ 10 were considered adherent^30^.

### Statistical analysis

IBM SPSS version 25 was used and continuous data was represented by the mean and standard deviation; categorical data was represented by the frequency and percentage (SD). Pearson's chi-square or Fisher exact tests were used to explore the connection between adherence to ART as a dependent variable and the independent variables. To identify food insecurity and other characteristics related with adherence to ART, a multivariate regression analysis was undertaken, starting with basic logistic regression with (p=0.25)^31^. Using an entry variable selection, multiple logistic regression with a 95% confidence interval was performed. The alpha 0.05 significance level was used.

## Results

There were 385 participants, 67.5% of whom were female and 32.5% were male. The majority of the respondents (46%) were between the ages of 41 and 50, and more than half of them (54.8%) were of Hausa ethnicity. Muslims made up the majority of the responders (70.6%). Over half of the respondents (57.7%) have a monthly income of more than 40,000 naira (US$97.23). About 23.1 percent of those polled were jobless or retired, 49.1% were self-employed or business owners, and 27.8% worked for the government. Over half of the respondents (60.3%) were married, while 19% were single and 20.8 percent were divorced or widowed. (Table 1).

**Table 1:** Distribution of respondents by Socio-Demographic Characteristics n=385

Variables	n	%	Mean ± SD
**Age (Years)**			40.77 ± 9.94
18–33	72	18.7	
34–48	117	30.4	
49–64	196	50.9	
**Gender**			
Male	125	32.5	
Female	260	67.5	
**Ethnicity**			
Hausa	211	54.8	
Yoruba	65	16.9	
Igbo	53	13.8	
Others	56	14.5	
**Religion**			
Christianity	113	29.4	
Islam	272	70.6	
**Educational level**			
No formal	32	8.3	
Primary school	66	17.1	
Secondary school	114	29.6	
Tertiary	173	44.9	
**Duration of treatment**			
6–12 months	129	33.5	
13–24 months	42	10.9	
25–36 months	97	25.2	
> 36months	117	30.4	
**Household size**			
1–4	162	42.1	
5–8	181	47.0	
9–12	35	9.1	
> 12	7	1.8	
**Monthly income**			59280 ± 56535.10
10000–20000 naira	77	20.0	
20001–30000 naira	64	16.6	
30001–40000 naira	22	5.7	
> 40000 naira	222	57.7	
**Occupation**			
Unemployed/retired	89	23.1	
Self-employed/business	189	49.1	
Government employed	107	27.8	
**Marital status**			
Single	73	19.0	
Married	232	60.3	
Widows and divorced	80	20.8	

*Others (From other ethnicity) **1 USD = 411.50 Naira,

### Prevalence of food security status and adherence to ART

More than two-thirds of the respondents (87%) were food insecure. Meanwhile, about 46 percent of the patients were non-adherent. (See Table 2)

**Table 2:** Prevalence of Food Security Status and Adherence to ART of the Respondents

Variables	Score	n	%
Food secure	0-1	50	13.0
Food insecure	2-6	335	87.0
Good adherence	>10	208	54.0
Poor adherence	<10	177	46.0

Table 3 outlines the important characteristics that influence adherence to ART. Adherence to ART was found to be significantly related to age (*X*^2^ = 9.179, *p* < 0.05). A greater proportion of ART non-adherents was found in respondents who were 49-64 years old (59.7%) compared to those who were 18-33 (38.9%) and 34-48 (53.8%) years old. Besides, non-adherence to ART was significantly associated with educational level (*X*^2^ = 8.458, *p* < 0.05). A higher proportion of ART non-adherence was found among respondents who had attained tertiary education (50.0%), compared to respondents who had attained no formal education (7.7%), primary (13.5%), and secondary education (28.8%). Occupation was associated with being adherent to ART (*X*^2^ = 9.061, *p* < 0.05). A greater proportion of non-adherents to ART were found among the government employed (60.7%) than the self-employed/ business (56.6%) and unemployed (40.4%). Marital status was also significantly associated with adherents to ART (*X*^2^ = 7.293, *p* < 0.05). Respondents who were divorced or widowed had a higher proportion of non-adherents to ART (66.3%) than those who were single (45.2%) and married (52.6%). Furthermore, respondents' food security status was linked to adherence to ART in a significant way (*p* < 0.05). A greater proportion of non-adherence was found among respondents who were food insecure (52.8%) than among respondents who were food secure (38.0%).

**Table 3:** Association between Socio-demographic, Food Insecurity and Adherence to ART

Variables	Adhere n (%)	Non- adhere n (%)	χ^2^	*p*-value
**Age (Years)**			9.179	**0.010**
18–33	44 (61.1)	28 (38.9)		
34–48	54 (46.2)	63 (53.8)		
49–64	79 (40.3)	117 (59.7)		
**Gender**			2.706	0.100
Male	65 (36.7)	60 (28.8)		
Female	112 (63.3)	148 (71.2)		
**Ethnicity**			2.865	0.413
Hausa	96 (54.2)	115 (55.3)		
Yoruba	25 (14.1)	40 (19.2)		
Igbo	28 (15.8)	25 (12.0)		
Others	28 (15.8)	28 (13.5)		
**Religion**			2.506	0.113
Christianity	59 (33.3)	54 (26.0)		
Islam	118 (66.7)	154 (74.0)		
**Educational level**			8.458	**0.031**
No formal	16 (9.0)	16 (7.7)		
Primary school	38 (21.5)	28 (13.5)		
Secondary school	54 (30.5)	60 (28.8)		
Tertiary	69 (39.0)	104 (50.0)		
**Duration of treatment**			0.530	0.912
6–12 months	60 (33.9)	69 (33.2)		
13–24 months	18 (10.2)	24 (11.5)		
25–36 months	47 (26.6)	50 (24.0)		
> 36months	52 (29.4)	65 (31.3)		
**Household size**			2.625	0.453
1–4	69 (39.0)	93 (44.7)		
5–8	91 (51.4)	90 (43.3)		
9–12	14 (7.9)	21 (10.1)		
> 12	3 (1.7)	4 (1.9)		
**Monthly income**			1.478	0.687
10000–20000 naira	37 (20.9)	40 (19.2)		
20001–30000 naira	27 (15.3)	37 (17.8)		
30001–40000 naira	8 (4.5)	14 (6.7)		
> 40000 naira	105 (59.3)	117 (56.3)		
**Occupation**			9.061	**0.011**
Unemployed	53 (59.6)	36 (40.4)		
Self-employed/business	82 (43.4)	107 (56.6)		
Government employed	42 (39.3)	65 (60.7)		
**Marital status**			7.293	**0.026**
Single	40 (54.8)	33 (45.2)		
Married	110 (47.4)	122 (52.6)		
Widows and divorced	27 (33.8)	53 (66.3)		
**Food security status**			11.446	**0.001**
Food secure	19 (62)	31(38)		
Food insecure	158 (47.2)	177 (52.8)		

Note: significant level at *p* < 0.05; ^b^Fishers exact test.

### Factors Associated with Adherence to ART

Table 4 shows that respondents who were self-employed were more likely to be ART non-adherents than those who were employed. When compared to unemployed participants, self-employed participants had a twofold increase in chances (AOR = 2.646, 95% CI: 1.335, 5.241). In addition, respondents who worked for the government had a 2.8-fold higher chance of not adhering to ART than those who did not (AOR = 2.842, 95% CI: 1.542, 5.240). Divorced or widowed respondents were also twice as likely as single or married respondents to stop using ART (AOR = 2.016, 95% CI: 1.111, 3.660). Those who were food insecure were also 1.2 times more likely than those who were food secure to be non-adherence to ART (AOR = 1.220, 95% CI: 1.642, 2.319).

**Table 4:** Factors associated to adherence to ART

Factors	B	SE	Adjusted OR	95CI	*p*-value
**Age (Years)**					
18–33	ref				
34–48	0.461	0.401	1.585	0.723–3.478	0.250
49–64	0.411	0.394	1.508	0.696–3.268	0.297
**Educational level**					
No formal	ref				
Primary school	-0.446	0.489	0.640	0.245–1.669	0.361
Secondary school	-0.197	0.450	0.821	0.340–1.983	0.661
Tertiary	0.093	0.438	1.098	0.465–2.592	0.832
**Occupation**					
Unemployed	ref				
Self-employed/business	0.973	0.349	2.646	1.335–5.241	**0.005**
Government employed	1.045	0.312	2.842	1.542–5.240	**0.001**

**Marital status**					
Single	ref				
Married	0.204	0.368	1.226	0.596–2.520	0.580
Others	0.701	0.304	2.016	1.111–3.660	**0.021**
**Food secure status**					
Food secure	ref				
Food insecure	0.199	0.328	1.220	1.642–2.319	**0.050**

Note: significant *p* < 0.05, S.E-standard error, Hosmer and Lemeshow test (*p* = 0.293), Cox and Snell R squared (18%), Nagelkereke R-squared (0.243), Omnibus Tests of Model Coefficients (*p* = 0.000).

## Discussion

In comparison to other studies conducted in Nigeria, the prevalence of adherence in this study is lower than the WHO's recommended limit^32^. Oku et al. found that 59.9% of patients in the south-south region of Nigeria were adhering to ART^1^ while Agu et al. found that 84.7% of patients in the north-central region of Nigeria were adhering to ART^12^. However, several Nigerian regions found decreased adherence. Because of inadequate educational status and medicine reminder technique, adherence was found to be 42% in Ekiti and Ondo states^24^ and 53.6% in Abuja, Nigeria's capital^33^. When compared to other regions in Nigeria, ART adherence was found to be lower among HIV respondents on ART in ABUTH, Zaria. The majority of patients interviewed in this study were non-adherent in aspects of forgetfulness and time, that could be attributed to a bad health sector and facilities, such as poor relationships with healthcare practitioners, long waiting times, and vast distance. These factors contributed to low adherence to ART in the current study compared to other studies done in other regions of Nigeria.

One of the characteristics affecting adherence to ART was respondents' profession. Government employees had a larger percentage of non-adherent respondents than self-employed/business owners or unemployed respondents, according to the current study. Nachega et al. observed that HIV-infected patients who have been employed were much more likely than those who were unemployed to fail to adhere to ART^11^. Non-adherence to ART was more common among self-employed respondents than among unemployed respondents. Other research, however, corroborated our findings, demonstrating a substantial connection between occupation and adherence to ART. Suryana et al. observed a strong relationship between ART adherence and job status^34^. According to Prah et al., occupation was proven to be a major factor in treatment adherence^35^. Compliance with ART was substantially associated to occupational position^36^. Non-adherence among HIV-positive employed or self-employed people was most frequently caused by busy work schedules and/or forgetfulness.

In this study, married people made up the majority of the respondents. Divorced or widowed respondents were assigned to the “other” category. Divorced and widowed respondents were two times more likely than married respondents to stop using ART. Previous research has discovered that marital status has a significant impact on ART adherence^37^-^40^. In their research, they discovered that marital status was a major determinant of adherence. Married expecting women are more likely to take ART than unmarried, formerly married, or cohabitating, according to their research^37^. Divorced adults had the lowest levels of adherence compared to other marital situations, with fewer women following the rules. Given that most married women had told their sex partners of their Infection status contributed significantly to their high adherence^37^. It indicated that spouses influenced HIV-positive people's adherence to treatment.; in other words, having a supporting partner can help enhance adherence to ART.

The majority of respondents (87%) were food insecure, according to the current survey. These final results beat studies performed at Arba Minch General Hospital in the Southern Region (19.5%), Kenya (20-50%), Uganda (37.3%), the Democratic Republic of the Congo (57%), Namibia (67%), Humera Hospital in Northern Ethiopia (40.4%), Jimma University Referral Hospital (63%), and Butajira Hospital inside the Southern Nation (87.4%). The current study found that food insecurity status was higher than other African countries. This might be due to the socioeconomic position of the respondents and the economic situation in the country, such as lack of job opportunities, poverty, and corruption. Differences in food insecurity levels between parts of the country can also be linked to the health intervention metrics employed, the research years and contexts, and the different indicators used to define food insecurity^41^-^46^.

Non-adherence to ART was 1.2 times more common in food needy respondents than in food secure respondents. This finding is consistent with that of a systematic review study, which indicated shortage of food is the most major barrier to regular ART adherence and the most common reason of prescription interruptions. Participants said they had greater unfavourable side effects when they used ART without food. Many individuals believed that if you don't eat adequate calories, the medicine will be ineffective or perhaps even harmful^47^. Patients who were food insecure had a higher rate of non-adherence to antiretroviral medication (ART) than those who had access to food. Noncompliance with ART has been linked to food scarcity effects in both developing and developed countries, according to research.

## Conclusion

In summary, food insecurity, occupation, and marital status were factors associated with adherence to ART among HIV adults. Workplace regulations must be in place to help those with illnesses in keeping to their medication regimens, and also self- employed patients can develop a self-reminder method like an alarm to reduce forgetfulness. Improved food shortages may also lead to higher adherence to treatment. To figure out how, intervention studies are required. It is critical to provide support for those living with HIV in order to solve ART adherence concerns among HIV-positive people. More adherence research studies should be undertaken on a regular basis to assess ART patients' levels of adherence and to prepare interventions to promote adherence. Adherence counsellors and adherence support should collaborate with the Regional Health Office, Regional HIV/AIDS Prevention Coordinating Office, and adherence counsellors. Government agencies and/or non-governmental organizations (NGOs) must maintain household food security for adults on ART by providing food assistance or offering job possibilities for those who are unemployed.

## References

[1] Oku AO, Owoaje ET, Oku OO, Monjok E (2013). Prevalence and determinants of adherence to highly active antiretroviral therapy (HAART) amongst a cohort of HIV positive women accessing treatment in a tertiary health Facility in Southern Nigeria. J HIV AIDS Infect Dis.

[2] Joint United Nations Programme on HIV/AIDS (UNAIDS) (2019). Global AIDS update 2019–communities at the centre.

[3] Steel G, Nwokike J, Joshi MP (2007). Development of a multi-method tool to measure ART adherence in resource-constrained settings: the South Africa experience. RPM Plus.

[4] Coetzee B, Kagee A, Vermeulen N (2011). Structural barriers to adherence to antiretroviral therapy in a resource-constrained setting: the perspectives of health care providers. AIDS care.

[5] Wagner GJ, Ryan GW (2004). Relationship between routinization of daily behaviors and medication adherence in HIV-positive drug users. AIDS Patient Care and STDs.

[6] Memiah P, Shumba C, Etienne-Mesubi M, Agbor S, Hossain MB, Komba P, Niyang M, Biadgilign S (2014). The effect of depressive symptoms and CD4 count on adherence to highly active antiretroviral therapy in sub-Saharan Africa. Journal of the International Association of Providers of AIDS Care (JIAPAC).

[7] Banagi Yathiraj A, Unnikrishnan B, Ramapuram JT, Kumar N, Mithra P, Kulkarni V, Holla R, Darshan B, Thapar R (2016). Factors influencing adherence to antiretroviral therapy among people living with HIV in Coastal South India. Journal of the International Association of Providers of AIDS Care (JIAPAC).

[8] Igbende AD, Dooior M, Ogwuche C (2016). Belief in spiritual healing, gender and adherence to medication among HIV/AIDS patients in Benue State, Nigeria. Int J Health Psychol Res.

[9] Mukui IN, Ng'ang'a L, Williamson J, Wamicwe JN, Vakil S, Katana A, Kim AA (2016). Rates and predictors of non-adherence to antiretroviral therapy among HIV-positive individuals in Kenya: results from the second Kenya AIDS indicator survey, 2012. PloS one.

[10] Calder Cedrina L (2020). “Adherence to Combination Antiretroviral Therapy among Pregnant Women Enrolled in a HIV Prevention Program in Rural North-central Nigeria.”. International journal of MCH and AIDS.

[11] Nachega JB, Uthman OA, Peltzer K, Richardson LA, Mills EJ, Amekudzi K, Ouédraogo A (2014). Association between antiretroviral therapy adherence and employment status: systematic review and meta-analysis. Bulletin of the World Health Organization.

[12] Agu KA, RC K, MA I, PG I (2011). Medication Adherence and Risk factors for non-adherence among patients taking Highly Active Antiretroviral Therapy. West African Journal of Pharmacy.

[13] Bello SI (2011). HIV/AIDS patients' adherence to antiretroviral therapy in Sobi Specialist Hospital, Ilorin, Nigeria. Glob J Med Res.

[14] Anyaike C, Atoyebi OA, Musa OI, Bolarinwa OA, Durowade KA, Ogundiran A, Babatunde OA (2019). Adherence to combined Antiretroviral therapy (cART) among people living with HIV/AIDS in a Tertiary Hospital in Ilorin, Nigeria. Pan African Medical Journal.

[15] Mûnene E, Ekman B (2015). Association between patient engagement in HIV care and antiretroviral therapy medication adherence: cross-sectional evidence from a regional HIV care center in Kenya. AIDS care.

[16] Lyimo RA, Stutterheim SE, Hospers HJ, de Glee T, van der Ven A, de Bruin M (2014). Stigma, disclosure, coping, and medication adherence among people living with HIV/AIDS in Northern Tanzania. AIDS patient care and STDs.

[17] Jones D, Cook R, Spence A, Weiss SM, Chitalu N (2014). Antiretroviral therapy in Zambia: do partners on ART enhance adherence?. Journal of the International Association of Prowders of AIDS Care (JIAPAC).

[18] Weiser SD, Young SL, Cohen CR, Kushel MB, Tsai AC, Tien PC, Hatcher AM, Frongillo EA, Bangsberg DR (2011). Conceptual framework for understanding the bidirectional links between food insecurity and HIV/ AIDS. The American journal of clinical nutrition.

[19] Goudge J, Ngoma B (2011). Exploring antiretroviral treatment adherence in an urban setting in South Africa. Journal of public health policy.

[20] Senkomago V, Guwatudde D, Breda M, Khoshnood K (2011). Barriers to antiretroviral adherence in HIV-positive patients receiving free medication in Kayunga, Uganda. AIDS care.

[21] Koethe JR, Blevins M, Bosire C, Nyirenda C, Kabagambe EK, Mwango A, Kasongo W, Zulu I, Shepherd BE, Heimburger DC (2013). Self-reported dietary intake and appetite predict early treatment outcome among low-BMI adults initiating HIV treatment in sub-Saharan Africa. Public health nutrition.

[22] Najjar A, Amro Y, Kitaneh I, Abu-Sharar S, Sawalha M, Jamous A, Qiq M, Makharzeh E, Subb Laban B, Amro W, Amro A (2015). Knowledge and adherence to medications among Palestinian geriatrics living with chronic diseases in the West Bank and East Jerusalem. PLoS One.

[23] Gugsa S, Potter K, Tweya H, Phiri S, Sande O, Sikwese P, Chikonda J, O'Malley G (2017). Exploring factors associated with ART adherence and retention in care under Option B+ strategy in Malawi: A qualitative study. PloS one.

[24] Afe AJ, Motunrayo O, Ogungbade GO (2018). Factors Influencing Adherence to HAART among Patients Living with HIV Infection in Southwest Nigeria: A CrossSectional Analysis. J HIV Retrovirus.

[25] Okunola OA, Muoghalu CO, Irinoye AI Socio-Cultural Factors Influencing Adherence to Antiretroviral Therapy among People Living with HIV/AIDS in Obafemi.

[26] NACA ‘National Strategic Framework on HIV and AIDS: (2017) 2017-2021’ [pdf].

[27] Rodger AJ, Cambiano V, Bruun T, Vernazza P, Collins S, Van Lunzen J, Corbelli GM, Estrada V, Geretti AM, Beloukas A, Asboe D (2016). Sexual activity without condoms and risk of HIV transmission in serodifferent couples when the HIV-positive partner is using suppressive antiretroviral therapy. Jama.

[28] Bickel G, Nord M, Price C, Hamilton W, Cook J Guide to measuring household food security.

[29] USDA (2008). U.S. Household Food Security Survey Module: Six-Item Short Form.

[30] Mannheimer SB, Mukherjee R, Hirschhorn LR, Dougherty J, Celano SA, Ciccarone D, Graham KK, Mantell JE, Mundy LM, Eldred L, Botsko M (2006). The CASE adherence index: A novel method for measuring adherence to antiretroviral therapy. AIDS care.

[31] Bendel RB, Afifi AA (1977). Comparison of stopping rules in forward “stepwise” regression. Journal of the American Statistical association.

[32] World Health Organization (2003). Adherence to long-term therapies: evidence for action.

[33] Avong YK Adherence to anti-retroviral therapy in the federal capital territory, Abuja, Nigeria.

[34] Aberman NL, Rawat R, Drimie S, Claros JM, Kadiyala S (2014). Food security and nutrition interventions in response to the AIDS epidemic: assessing global action and evidence. AIDS and Behavior.

[35] Musumari PM, Wouters E, Kayembe PK, Kiumbu Nzita M, Mbikayi SM, Suguimoto SP, Techasrivichien T, Lukhele BW, El-Saaidi C, Piot P, Ono-Kihara M (2014). Food insecurity is associated with increased risk of non-adherence to antiretroviral therapy among HIV-infected adults in the Democratic Republic of Congo: a cross-sectional study. PloS one.

[36] Hong SY, Fanelli TJ, Jonas A, Gweshe J, Tjituka F, Sheehan HM, Wanke C, Terrin N, Jordan MR, Tang AM (2014). Household food insecurity associated with antiretroviral therapy adherence among HIV-infected patients in Windhoek, Namibia. J Acquir Immune Defic Syndr.

[37] Mensa M, Belijo Z N (2017). Levels and Predictors of Food Insecurity among HIV Positive Adult Patients Taking Highly Active Anti-Retroviral Therapy at Arba Minch General Hospital, Southern Ethiopia, 2016. General Medicine: Open Access.

[38] Gebre BB (2019). Factors associated with alcohol use disorder among people living with HIV/AIDS attending art Clinic, Mizan Tep University teaching hospital, south West Ethiopia. HIV/AIDS (Auckland, NZ).

[39] Oluma A, Abadiga M, Mosisa G, Etafa W, Fekadu G (2020). Food Insecurity among people living with HIV/AIDS on ART follower at public hospitals of Western Ethiopia. International Journal of Food Science.

[40] Chop E, Duggaraju A, Malley A, Burke V, Caldas S, Yeh PT, Narasimhan M, Amin A, Kennedy CE (2017). Food insecurity, sexual risk behavior, and adherence to antiretroviral therapy among women living with HIV: a systematic review. Health care for women international.

[41] Suryana K, Suharsono H, Antara IG (2019). Factors associated with adherence to anti-retroviral therapy among people living with HIV/AIDS at Wangaya Hospital in Denpasar, Bali, Indonesia: A Cross-Sectional Study. Hiv/aids (Auckland, NZ).

[42] Prah J, Hayfron-Benjamin A, Abdulai M, Lasim O, Nartey Y (2018). Factors affecting adherence to antiretroviral therapy among HIV/AIDS patients in cape coast metropolis. Ghana J HIV AIDS.

[43] Safira N, Lubis R, Fahdhy M (2018). Factors affecting adherence to antiretroviral therapy. KnE Life Sciences.

[44] Adeniyi OV, Ajayi AI, Ter Goon D, Owolabi EO, Eboh A, Lambert J (2018). Factors affecting adherence to antiretroviral therapy among pregnant women in the Eastern Cape, South Africa. BMC infectious diseases.

[45] Bam K, Rajbhandari RM, Karmacharya DB, Dixit SM (2015). Strengthening adherence to Anti Retroviral Therapy (ART) monitoring and support: operation research to identify barriers and facilitators in Nepal. BMC health services research.

[46] Odili VU, Obieche AO, Amibor KC (2017). Adherence to antiretroviral therapy and its determinants among HIV-infected patients in Nigeria. Journal of pharmacy practice.

[47] Ekama SO, Herbertson EC, Addeh EJ, Gab-Okafor CV, Onwujekwe DI, Tayo F, Ezechi OC (2012). Pattern and determinants of antiretroviral drug adherence among Nigerian pregnant women. Journal of pregnancy.

